# The mothers’ breastfeeding behaviour within six weeks postpartum: new scale development and psychometric validation study

**DOI:** 10.1186/s12884-023-05439-2

**Published:** 2023-03-02

**Authors:** Jing-Ling Wu, Shu-Qin Pang, Xiu-Min Jiang, Yan Lin, Qing-Xiang Zheng

**Affiliations:** 1 School of Nursing, Fujian University of Traditional Chinese Medicine, Fuzhou, Fujian China; 2 Fujian Maternity and Child Health Hospital College of Clinical Medicine for Obstetrics & Gynecology and Pediatrics, Fujian Medical University, Fuzhou, Fujian China

**Keywords:** Mother, Breastfeeding, Behaviour, Six weeks postpartum, Scale, Reliability, Validity

## Abstract

**Background:**

The evaluation of mothers’ breastfeeding behaviour within 6 weeks postpartum could help health workers comprehensively identify maternal breastfeeding shortcomings, clarify nursing problems, and provide targeted interventions. However, no prior study was found, therefore this study aimed to develop and validate the reliability and validity of the mothers’ breastfeeding behaviour scale within 6 weeks postpartum.

**Methods:**

A main two-step approach was used: (1) a qualitative pilot study using the purposive sampling method was adopted to test the fitness, simplicity, and clarity of items with 30 mothers; (2) a cross-sectional survey using the convenient sampling method was conducted for item analysis and psychometric validation with 600 mothers.

**Results:**

The final version of the scale consisted of 36 items with seven dimensions, explaining 68.852% of the total variance. The Cronbach’s α, split-half, and retest coefficients were 0.958, 0.843, and 0.753, respectively. The validity of the scale: (1) Content validity: content validity index (CVI) range of items was between 0.882 and 1.000. The scale-level-CVI was 0.990. (2) Structure validity: The fitting indices were as follows: *χ*^2^/ⅆ*f* =2.239, RMR = 0.049, RMSEA = 0.069, TLI = 0.893, CFI = 0.903, IFI = 0.904, PGFI = 0.674, and PNFI = 0.763. (3) Convergent validity: The composite reliability and average variance extracted (AVE) of the seven dimensions were between 0.876 and 0.920 and between 0.594 and 0.696. (4) Distinguish validity: The correlation coefficients were less than the square root of the AVE, except for self-decision behaviour, self-coping behaviour, and self-control behaviour. However, the fit index of the original three-factor model was better than that of the other new models, with significant differences (*P* < 0.001). (5) Calibration validity: The area under the curve was 0.860 or 0.898 when the scale was used to predict exclusive or any breastfeeding at 42 days. The correlation coefficients of the maternal breasting feeding evaluation scale, breastfeeding self-efficacy short-form scale, and the scale were 0.569 and 0.674, respectively.

**Conclusion:**

The newly developed mothers’ breastfeeding behaviour scale within 6 weeks postpartum consists of 36 items belonging to seven dimensions with good reliability and validity and is a reliable and valid instrument to be used in future maternal breastfeeding behaviour assessments and interventions.

## Background

Breastfeeding protects mothers against breast cancer and cardiovascular diseases in later life [1, 2], offers infants better mental and intellectual development [3], strengthens mother-infant bonding, and saves related health care costs [4]. The World Health Organization, among other institutions, recommends that all infants receive exclusive breastfeeding until 6 months postpartum and sets the goals for achieving a six-month exclusive breastfeeding rate of at least 50% in 2025 [5]. However, despite substantial evidence and many policies indicating that breastfeeding is a healthy behaviour for infants, mothers, and society, recent estimates show that the Chinese exclusive breastfeeding rate at 6 months is 29.5%, and there is a 30.71% country six-month exclusive breastfeeding rate in the world at less than 20%, which is a significant gap from the WHO targe [6, 7]. Therefore, effectively increasing the exclusive breastfeeding rate under 6 months is a common problem that many countries and international organizations need to solve together. Many articles have discussed the exclusive breastfeeding rate, and a rule was summarized that the initial breastfeeding incidence is higher and drops sharply after being discharged from the hospital over time [8, 9]. Recently, a survey was conducted in which 67.9% of mothers stopped exclusive breastfeeding within the first 6 weeks postpartum among mothers who gave up under the first 6 months, which emphasized the significance of 6 weeks postpartum and provided a unique perspective to upgrade the exclusive breastfeeding rate [10].

The first 6 weeks postpartum, also known as puerperium, is a variable and particular period for maternal psychic recovery and social and emotional modifications [11]. The birth of a newborn breaks the original balance and pushes forward women to experience role changes, complete role adaptation, and finally achieve maternal role attainment [12]. In the process, the woman needs to play the new role of mother and take responsibility for meeting expectations given by society, such as breastfeeding [13]. Six weeks postpartum is the transition period for breastfeeding, and the transition theory proposed by Professor Meleis defines the transition as a process from one stable state to another stable state when needs change, with an unstable phase in the midst [14]. To achieve breastfeeding, mothers within 6 weeks postpartum need to form new stable behaviour patterns to replace actual behaviour by learning breastfeeding knowledge and skills, coping with breastfeeding challenges, and adjusting negative emotions [15, 16]. The new behaviour pattern may appear as an inherent behavioural feature that affects maternal cognition, decision-making or action on breastfeeding during the 6 months postpartum or even the subsequent pregnancy [17]. However, due to mothers’ own physical or/and psychological vulnerabilities and insufficient support from health professionals within 6 weeks postpartum, mothers are prone to behavioural disorders and eventually develop an ineffective breastfeeding behaviour pattern, resulting in poor breastfeeding conditions [18, 19]. Hence, our research team deems that exploration of breastfeeding behaviour within 6 weeks postpartum is one of the breakthroughs to promote breastfeeding practice.

The term breastfeeding behaviour is widely used, but it is a summary concept for breastfeeding mode and specific breastfeeding technologies and not yet a distinct concept in the literature, making it difficult to operationalize [20–23]. Moreover, to our knowledge, mothers’ breastfeeding behaviour within 6 weeks postpartum has not yet received attention from other researchers. In our previous research, we were inspired by the COM-B system, transitions theory, maternal role attainment theory, and related literature to complete the conceptual analysis [24–29]. The mothers’ breastfeeding behaviour within 6 weeks postpartum was defined as mothers performing breastfeeding psychological reactions or movements depending on the internal regulation of their own capability, motivation and opportunity under external stimulation from the social environment, social resources, and infant behaviour, and its attributes include self-regulation behaviour, resource utilization behaviour, and at-the-breast feeding behaviour. A scientific and practical instrument is necessary for subsequent relevant research, which is required to accurately identify behavioural shortcomings and nursing problems to provide practical and targeted support for mothers within 6 weeks postpartum. Compared with other indicators, the observation and evaluation of maternal breastfeeding behaviour is more intuitive and direct, which is more valuable for nurses and midwives to comprehensively identify potential shortcomings and provide targeted interventions. However, these existing scales focus on measuring why mothers give up breastfeeding early, such as breastfeeding knowledge, skills, attitudes, satisfaction, self-efficacy, and competency [6, 30–34]. No prior study was found to develop a scale for evaluating breastfeeding behaviour, especially for mothers within 6 weeks postpartum, hence warranting careful study. To fill this gap, this study aimed to develop the mothers’ breastfeeding behaviour scale within 6 weeks postpartum (MBBC-6 W) and validate its reliability and validity to provide an assessment tool for related evaluation and intervention in future research.

## Methods

### Design and setting

A sequential exploration mixed-method study was carried out in the departments of obstetrics of Fujian Maternity and Child Health Hospital, a tertiary and baby-friendly specialist hospital in Fuzhou, Fujian province, P.R. of China. This scale development and scale validation study was implemented by a qualitative pilot study (Step one) and a formal cross-sectional investigation (Step two). Step one collected the reaction and suggestions of the target population to verify face validity by a face-to-face structured interview. Step two involved conducting a cross-sectional survey with the convenience sampling method to analyse item performance and finish psychometric validation.

### Population and recruitment

Two samples were used to implement the development and validation in this study. The first sample (Sample one) was collected to understand whether the designed items were fitness, simplicity, and clarity in the target population of mothers within 6 weeks postpartum. After generating a list of postpartum women from medical records, the purposive sampling strategy was employed to ensure that the sample was diverse in terms of parity, delivery mode, education level, and infant whereabouts. Thirty mothers within 6 weeks postpartum who met the inclusion criteria were interviewed to participate in the pilot study from July 1, 2020, to July 15, 2020 [35]. The second sample (Sample two) was collected to encompass the item analysis to form the final scale version and finish psychometric validation by three key steps: (1) sampling, the potential participants who delivered women in the obstetrics department were randomly recruited in one interaction with each mother separately between July 22, 2020, and October 7, 2020. (2) introducing, the author provided mothers with the information on the purpose of the survey, the process of data collection, and the right to anonymity, confidentiality, and withdrawal at any time. (3) Screening: Potential participants who were interested in participating in this study were interviewed to sign an informed consent form and complete a final checklist for determining whether they met the inclusion criteria prior to starting the investigation via further communication. Regarding previous studies, 10–15 participants per item were necessary for construct validity, and 10–20% invalid questionnaires should be considered [6]. Therefore, the final calculated sample size interval was between 516 and 774, and the research group made the decision that 600 mothers were recruited for informal investigation. The inclusion criteria of sample one and sample two were as follows: (1) mothers within 6 weeks postpartum have a singleton, full-term, and healthy infant (1 min Apgar score > 8), (2) mothers feed their infant with breastfeeding, (3) the age of mother ≥20 years old, and (4) mothers had attended middle school or above and could communicate in Chinese generally. Mothers were excluded from the study if (1) women were experiencing mother-infant separation, (2) women-infant dyads who were prohibited from exclusive breastfeeding by the professional health provider due to medical conditions or other illnesses, (3) infants suffered from a severe disease, or (4) mothers had serious childbirth complications, emotional disorders, or cognitive impairment.

### Data collection and study assessment

For the pilot study, an author distributed the MBBC-6 W (version-I) and interviewed participants to express their perspectives concerning the scale item, and maternal reactions and suggestions were recorded. An initial consensus solution between mothers and investigators was proposed, which could provide a reference to form subsequent item generation of MBBC-6 W (version II) in the following group discussion. For the formal investigation, an author invited eligible participants to complete the investigation as follows: (1) add the WeChat or other messaging method with the consent of participants, (2) invite participants to select a time in the four time periods of “3-10 days, 11-19 days, 20-29 days, 30-42 days” to finish the questionnaire, (3) sent the personal information form, MBBC-6 W (version II), Maternal Breastfeeding Evaluation Scale (MBFES), and Breastfeeding Self-Efficacy Short Form Scale (BSES-SF) online at the appointed time, (4) 30 participants were randomly selected to fill out the MBBC-6 W (version II) again 2 weeks after completing the questionnaire, and (5) the feeding mode questionnaire was sent at 6 weeks postpartum.

In this study, six research questionnaires were used for data collection. (1) The first questionnaire, version-I of MBBC-6 W, was formed by the Delphi method and qualitative content analysis in our previous research [36], which is a self-reported scale with 45 items evaluating three categories: self-regulation behaviours, resource utilization behaviours, and at-the-breast feeding behaviours. For each item investigated, there is a score according to a five-point Likert scale (1 = it is strongly true about me, 2 = it is true about me, 3 = uncertainly, 4 = it is false about me, 5 = it is strongly false about me). The score ranges from 45 to 225, with higher scores indicating behaviours that are more conducive to breastfeeding. (2) The personal information form designed by the research team included maternal sociodemographic questions such as maternal age, marital status, education, monthly income, ethnicity, occupation, maternity leave, parity, and delivery mode. (3) The version II of MBBC-6 W, the updated version following group discussions after completing the pilot study. (4) The MBFES was designed to measure maternal perception of breastfeeding experience belonging to three dimensions: maternal enjoyment/role attainment, infant satisfaction/growth, and lifestyle/maternal body image [33]. The Chinese version of the MBFES was used as one of the calibration questionnaires in this study, which consists of 29 items with a Cronbach’s α coefficient of 0.952 [37]. (5) The BSES-SF, another calibration questionnaire, is a 14-item scale with a Cronbach’s α coefficient of 0.927 to evaluate maternal breastfeeding confidence and predict feeding mode at 4 and 8 weeks postpartum [34, 38]. (6) The last questionnaire was the feeding mode questionnaire, which consists of one question, “*How did you feed your baby between the first six months postpartum*”. Seven responses were set based on the breastfeeding definition from the United Nations Children’s Fund [39].

### Data analysis

The data were managed and analysed using SPSS 25.0, Amos 25.0, and Stata 15.1 software. The results of the pilot study and item performance verification were analysed to develop the final version scale. Then, the reliability and validity were verified to test the psychometric validation relying on the data of the cross-sectional investigation. All continuous data are presented as the mean-standard ($\overline{x}\pm s$), and categorical variables are presented as frequencies or percentages. In the stage of scale development, the research group discussed the suggestions and initial consensus formed by the participants and investigator in the pilot study and revised them one by one to form a new version of MBBC-6 W (version II). Then, the coefficient of variation (CV) method, critical ratio (CR) method, Cronbach’s α coefficient method, correlation coefficient method, and exploratory factor analysis (EFA) were used for item analysis one by one. The items that met the above five screening methods were retained, and the selection criteria were as follows [6]: (1) The value of CV, calculated as CV= $\overline{x}/s$, was used to judge the sensitivity of the item, that is, the ability to distinguish between different individuals. The item with CV < 0.15 was deleted. (2) The CR was used to test whether the item could distinguish the high group (top 27%) and low group (bottom 27%). If the CR value was less than 3.00 or there was no statistically significant difference (*P* > 0.05), the item was deleted. (3) The Cronbach’s α coefficient of the remaining items was calculated when the items were deleted one by one. Moreover, the item that caused a significant change in the coefficient was deleted. (4) The Pearson correlation method was used to calculate the correlation coefficient between items and items, corresponding dimensions, and scales. If the coefficient between two items had a high value (> 0.8) and the coefficient of the item and its dimension or scale was less than 0.4 or no significant difference (*P* > 0.05), the item was deleted. (5) The Kaiser–Meyer–Olkin (KMO) test (> 0.50) and the Bartlett test of sphericity (*P* < 0.05) were estimated to verify whether the scale was suitable for factor analysis. An item with commonality < 0.4, factor loading values < 0.4, or factor loading on different common factors ≥0.4 was deleted.

In the stage of scale validation, Cronbach’s α coefficient, split-half reliability, and retest reliability were used to verify the scale’s reliability, and their fitting criteria were 0.70, 0.80, and 0.70, respectively [6, 39]. The content validity, structure validity, convergence validity, distinguish validity, and calibration validity were used to verify the validity of this scale. (1) Content validity refers to whether the scale could measure the content of the target variable, which was assessed by the item-level content validity index (I-CVI) and average scale-level CVI (S-CVI/Ave) based on the experts’ importance score, and their qualified values were 0.8 and 0.9 [40]. (2) Structure validity refers to the fitting degree between the actual structure and the assumed structure, and it was verified by EFA and confirmatory factor analysis (CFA). In the EFA, principal axis factor analysis extracted the common factors, and promax rotation was used to obtain the factor loading matrix. The standard for common factor extraction is as follows [41]: (a) eigenvalues> 1.0, (b) scree plot, (c) cumulative variance contribution rate > 50%, and (d) the number of items contained in any common factor ≥ 3 [6]. Meanwhile, to make up for the methodological defects of scree plots, parallel analysis (PA) and minimum average partial correction (MPA) were used to determine the final number of common factors. In the CFA, considering the feature of each fitting index, the following index was adopted to assess the fitting effect: the normed chi-square (χ^2^/df), root mean square residual (RMR), root mean square error of approximation (RMSEA), Tucker–Lewis index (TLI), comparative fit index (CFI), incremental fit index (IFI), parsimony goodness-of-fit index (PGFI), and parsimony adjusted normed fit index (PNFI). If χ^2^/df ≤ 3, RMR < 0.05, RMSEA< 0.08, TLI > 0.90, CFI > 0.90, IFI > 0.90, PGFI> 0.50, and PNFI> 0.50, the model had a good fit. Among the 526 valid questionnaires, numbers 1 to 263 were used for EFA, and numbers 264 to 526 were used for CFA. (3) Convergence validity was used to determine the observed variable and cohesion of its dimension, assessed by standardized factor loadings, composite reliability (CR), and average variance extraction (AVE), and their threshold levels were 0.5, 0.6 and 0.5, respectively [42, 43]. (4) Distinguishing validity is the ability to distinguish different dimensions, tested by comparing the value of AVE and the determination coefficient (r^2^). If the value of r^2^ was less than the square root of the AVE, the model was regarded as having good distinguishing validity [44]. (5) Calibration validity was the correlation between scale scores and calibration scores, consisting of predictive validity and correlation validity. Receiver operating characteristic (ROC) analysis was used to test whether MBBC-6 W could predict exclusive breastfeeding or any breastfeeding at 42 days. An area under the ROC curve (AUC) greater than 0.7 was considered to indicate predictive validity [45]. In addition, the correlation coefficients of the MBFES, BSES-SF, and MBBC-6 W were calculated to verify the correlation validity, and the acceptable value was 0.5 [46].

## Results

### Participant sociodemographic characteristics

**Fig. 1:**
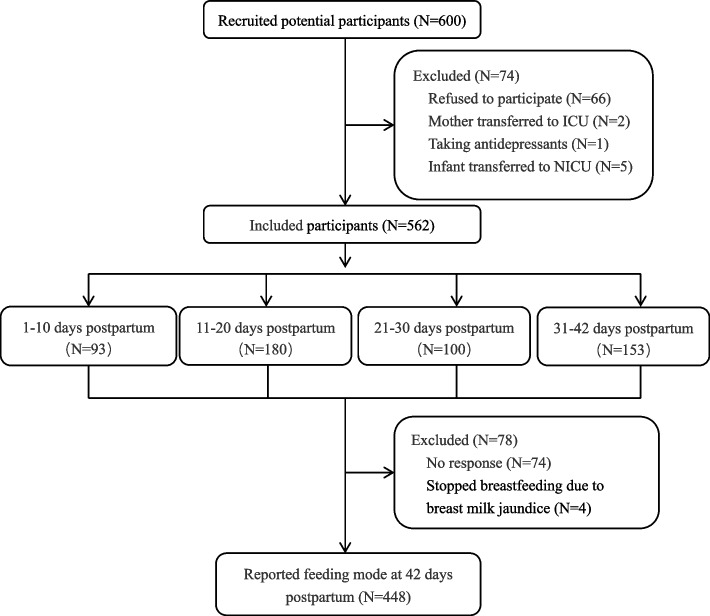
The detailed flowchart of included participants

In the pilot study, all 30 invited mothers agreed to participate in this study, ranging from 24 to 36 years old, with a mean of 30 ± 3.06 years old. Participants covered different education levels of middle school (3 cases), high school (5 cases), college (18 cases), master and above (7 cases), delivery mode of vaginal childbirth (17 cases) and caesarean section (13 cases), parity of primipara (16 cases) and multipara (14 cases), whereabouts of rooming-in (17 cases) and neonatal observation unit (13 cases). In the formal investigation, 562 mothers completed the questionnaire in the 600 recruited potential participants with a response rate of 87.67%, and 448 mothers finally reported the feeding mode at 6 weeks postpartum with a response rate of 79.72%. The detailed flowchart is shown in Fig. 1. The sociodemographic information of the included participants is reported in Table 1.

**Table 1:** The socio-demographic information of included participants (*N* = 526)

Items	N (%)
Age (years)	
< 35	441 (83.84)
≥ 35	85 (16.16)
Marital status	
Married	514 (97.72)
Unmarried	10 (1.90)
Other	2 (0.38)
Education	
Junior high school	40 (7.60)
High school	68 (12.93)
College	337 (70.92)
Master and above	45 (8.56)
Monthly income (yuan)	
< 5000	48 (9.13)
5000–9999	186 (35.36)
10,000–14,999	153 (29.09)
≥ 15,000	139 (26.43)
Ethnicity	
Han nationality	518 (98.48)
Other minorities	8 (1.52)
Occupation	
Full-time mother	173 (32.89)
Full-time job	319 (60.65)
Part-time job	34 (6.46)
Maternity leave	
< 42 days	11 (2.09)
42 days-6 months	269 (51.14)
> 6 months	246 (46.77)
Parity	
Primipara	295 (56.08)
Multipara	231 (43.92)
Delivery mode	
Vaginal childbirth	338 (64.26)
Cesarean section	188 (35.74)

### Scale development

In the pilot study, 22 mothers thought all items of MBBC-6 W (version I) were understandable and simple, and eight mothers proposed their confusion and suggestion for six items, mainly related to the problem of semantic expression. Based on the comments and the initial solution that reached a consensus between the investigator and mother, the research group revised the controversial items without changing the evaluation purpose. The expression of two items was modified, and four items were merged into two items. After this phase, version II of the MBBC-6 W was formed, consisting of 43 items scored on a 5-point Likert scale. The results of the item analysis for MBBC-6 W (version II) are shown in Table 2. Item 14 was excluded because the CV value was less than 0.15, and all the remaining items met the retention standards of the CV value, CR value, and Cronbach’s α coefficient test. The results of the correlation coefficient method suggested that the item-item correlations were between 0.074 and 0.775, the item-corresponding dimension correlations were between 0.566 and 0.886, and the item-scale correlations were between 0.295 and 0.754, in which the correlation coefficient of Item 3 and the scale score (*r* = 0.295) did not pass the preset standard. Then, a total of six rounds of EFA were performed, and all of them passed the Bartlett test (*P* < 0.001) and KMO test, ranging from 0.926 to 0.928. Three items (27, 28, 32) were excluded due to the commonality of less than 0.4, and two items (41, 43) were deleted because of the cross-loading in different common factors. After deleting the above five items, the factor loading and commonality of the remaining 36 items in the final version of MBBC-6 W reached the retention standard, as shown in Table 2.

**Table 2:** The result of items analysis for the MBBC-6 W (version-II)

Item	CV	CR	Cronbach’s α	Correlation coefficient		The last EFA result	
				Dimensions	Scale	Loading	Commonality
1	0.160	10.540^***^	0.959	0.820^**^	0.534^**^	0.782	0.679
2	0.160	11.551^***^	0.958	0.813^**^	0.587^**^	0.868	0.594
3	0.190	5.365^***^	0.960	0.566^**^	0.295^**^	NA	NA
4	0.160	12.119^***^	0.958	0.813^**^	0.634^**^	0.707	0.709
5	0.220	15.658^***^	0.958	0.835^**^	0.652^**^	0.821	0.677
6	0.250	14.266^***^	0.958	0.835^**^	0.646^**^	0.767	0.731
7	0.270	19.569^***^	0.958	0.805^**^	0.691^**^	0.494	0.604
8	0.200	18.409^***^	0.958	0.873^**^	0.739^**^	0.676	0.699
9	0.190	15.998^***^	0.958	0.860^**^	0.609^**^	0.827	0.719
10	0.190	14.444^***^	0.958	0.819^**^	0.635^**^	0.688	0.615
11	0.250	16.750^***^	0.958	0.794^**^	0.617^**^	0.550	0.727
12	0.200	19.865^***^	0.958	0.802^**^	0.697^**^	0.503	0.684
13	0.170	19.934^***^	0.958	0.830^**^	0.745^**^	0.583	0.425
14	0.110	NA	NA	NA	NA	NA	NA
15	0.190	16.844^***^	0.958	0.809^**^	0.655^**^	0.651	0.539
16	0.170	14.966^***^	0.959	0.800^**^	0.579^**^	0.889	0.604
17	0.170	14.324^***^	0.958	0.782^**^	0.584^**^	0.687	0.619
18	0.160	13.122^***^	0.959	0.801^**^	0.569^**^	0.600	0.521
19	0.190	16.349^***^	0.958	0.851^**^	0.658^**^	0.736	0.570
20	0.200	14.576^**^	0.958	0.824^**^	0.611^**^	0.616	0.482
21	0.220	16.941^***^	0.958	0.886^**^	0.667^**^	0.781	0.590
22	0.190	13.402^***^	0.958	0.835^**^	0.608^**^	0.738	0.466
23	0.170	14.874^**^	0.958	0.825^**^	0.673^**^	0.482	0.620
24	0.170	13.285^***^	0.959	0.833^**^	0.555^**^	0.877	0.769
25	0.200	18.843^***^	0.958	0.861^**^	0.700^**^	0.735	0.568
26	0.220	15.685^***^	0.958	0.858^**^	0.607^**^	0.854	0.547
27	0.180	14.494^***^	0.959	0.581^**^	0.539^**^	NA	NA
28	0.350	12.228^***^	0.960	0.641^**^	0.488^**^	NA	NA
29	0.220	15.420^***^	0.958	0.806^**^	0.602^**^	0.868	0.721
30	0.230	14.994^***^	0.958	0.816^**^	0.591^**^	0.869	0.591
31	0.200	17.032^***^	0.958	0.796^**^	0.649^**^	0.570	0.457
32	0.250	12.865^***^	0.959	0.640^**^	0.471^**^	NA	NA
33	0.210	16.587^***^	0.958	0.769^**^	0.620^**^	0.642	0.770
34	0.180	17.700^***^	0.958	0.735^**^	0.614^**^	0.613	0.619
35	0.180	17.733^***^	0.958	0.738^**^	0.620^**^	0.492	0.473
36	0.340	10.812^***^	0.960	0.698^**^	0.462^**^	0.777	0.717
37	0.260	19.195^***^	0.958	0.831^**^	0.646^**^	0.935	0.668
38	0.250	20.894^***^	0.958	0.865^**^	0.686^**^	0.864	0.490
39	0.220	18.546^***^	0.958	0.774^**^	0.623^**^	0.565	0.624
40	0.220	21.859^***^	0.958	0.810^**^	0.703^**^	0.548	0.619
41	0.260	18.780^***^	0.958	0.758^**^	0.656^**^	NA	NA
42	0.240	21.296^***^	0.958	0.792^**^	0.742^**^	0.505	0.629
43	0.180	17.236^***^	0.958	0.699^**^	0.690^**^	NA	NA

*NA* Not Available

^**^P < 0.01

^***^P < 0.001

### Scale validation

**Fig. 2:**
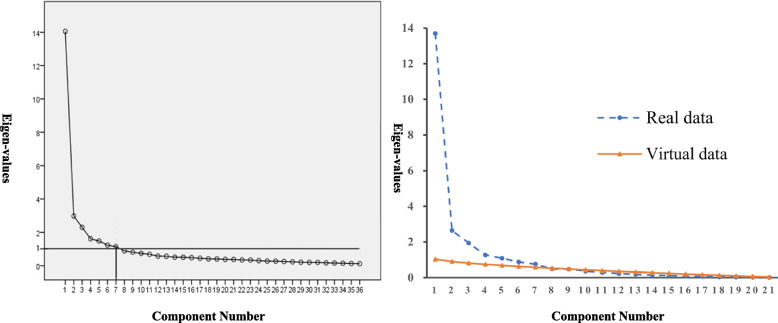
The scree plot of the sixth EFA (Left) and the result of PA (Right)

**Fig. 3:**
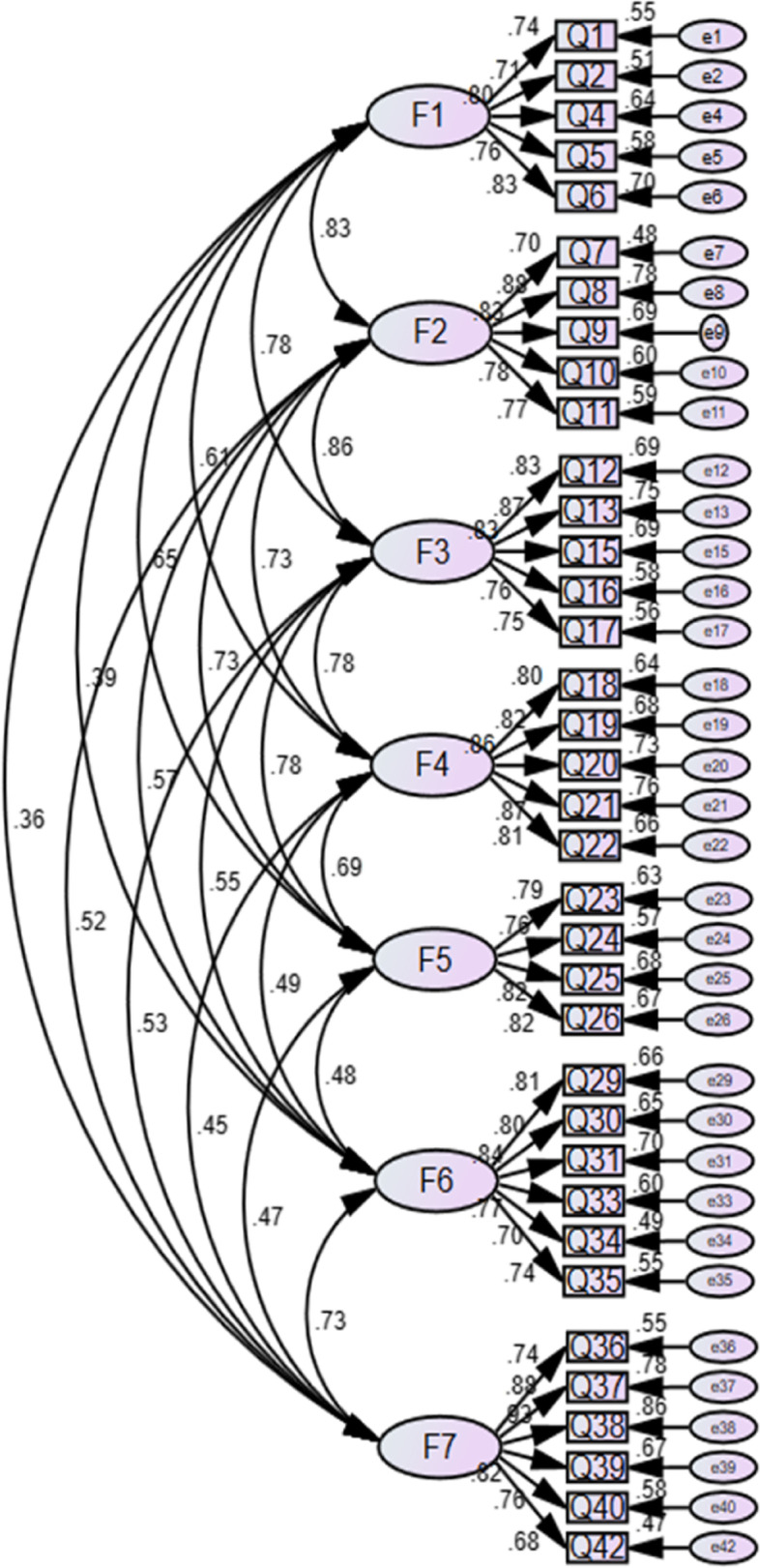
The standardized path diagrams of CFA

The Cronbach’s α coefficient, split-half coefficient, and retest coefficient for the final version of MBBC-6 W were 0.958, 0.843, and 0.753, respectively. The results of the validity test are as follows: (1) Content validity: According to the important score from 17 experts, the range of I-CVI values in this scale was between 0.882 and 1.000. For the scale level, the S-CVI/Ave value for the scale was 0.990, and the values for the three subscales were between 0.976 and 1.000. (2) Structure validity: The EFA results showed that the scree plot began to level off after the seventh factor, the PA showed that the eigenvalue of the eighth factor was smaller than that of the virtual data, and the MPA analysis showed that the square value of the average partial correction coefficient was the minimum when seven factors were extracted, as shown in Fig. 2. The above results indicated that seven factors should be extracted in line with the original assumptions. According to the features of each factor, seven factors were named self-decision behaviour (F1), self-coping behaviour (F2), self-control behaviour (F3), resource coordination behaviour (F4), resource acquisition behaviour (F5), breastfeeding operation skills (F6), and breastfeeding self-perception (F7). The standardized path diagram of MBBC-6 W is shown in Fig. 3. The seven-factor model accounted for 68.852% of the total variance, and each factor contained at least four items. The CFA results showed that the seven-factor model has an acceptable fit, with χ^2^/df = 2.239 (χ^2^ = 1283.15, df = 573), RMR = 0.049, RMSEA = 0.069, TLI = 0.893, CFI = 0.903, IFI = 0.904, PGFI = 0.674, and PNFI = 0.763. (3) Convergent validity: Fig. 3 shows the standardized factor loadings for each item in this scale, ranging from 0.84 to 0.93, whereby all values surpassed the recommended values. The CR values on seven factors from F1 to F7 were 0.879, 0.894, 0.904, 0.920, 0.876, 0.903, and 0.917. Correspondingly, the AVE values from F1 to F7 were 0.594, 0.629, 0.654, 0.696, 0.638, 0.609, and 0.650. (4) Distinguish validity: The result of the AVE method showed that, except for F1, F2, and F3, the value of r^2^ was less than the square root of the AVE, as shown in Table 3. Considering that the above three secondary dimensions belong to the same primary dimension, the chi-square difference test was used to further verify the distinguishing validity among them. The three factors were combined to form four new models: Model 1 (F1 and F2, F3), Model 2 (F1 and F3, F2), Model 3 (F2 and F3, F1), and Model 4 (F1, F2 and F3), which were compared with the original three-factor model on the fitting index. The results showed that the original model had the best fit and a significant difference in the χ2 value (*P* < 0.001), as shown in Table 4. (5) Calibration validity: The crude AUC was 0.860 when the scale was used to predict exclusive breastfeeding at 42 days postpartum, and the AUC was 0.857 after adjusting for parity, delivery mode, and postpartum infant whereabouts. The crude AUC was 0.898 when the scale was used to predict any breastfeeding at 42 days postpartum. Since only two primiparas did not feed their infants with breastfeeding, only two covariates of delivery method and infant whereabouts were adjusted, and its adjusted AUC was 0.903. Furthermore, the correlation coefficients of MBBC-6 W, MBFES, and BSES-SF were 0.569 and 0.674, respectively.

**Table 3:** The result of the AVE method for verifying distinguish the validity of the scale

Factors	F1	F2	F3	F4	F5	F6	F7
F1							
F2	0.828						
F3	0.777	0.857					
F4	0.613	0.725	0.785				
F5	0.650	0.732	0.781	0.685			
F6	0.390	0.574	0.555	0.489	0.477		
F7	0.363	0.522	0.531	0.450	0.470	0.727	
$\sqrt{AVE}$	0.771	0.793	0.809	0.834	0.799	0.780	0.806

$\sqrt{AVE}$ = the square root of the average variance extraction

**Table 4:** The result of the Chi-square test for verifying distinguish the validity of F1, F2, and F3

Model	*χ*^2^	*df*	*χ*^2^/*df*	RMR	RMSEA	TLI	CFI	IFI	PGFI	PNFI
Original model	1283.15	573	2.239	0.049	0.069	0.893	0.903	0.904	0.674	0.763
Model 1^***^	1417.89	579	2.449	0.520	0.074	0.875	0.886	0.886	0.661	0.755
Model 2^***^	1478.16	579	2.553	0.520	0.077	0.878	0.867	0.798	0.648	0.748
Model 3^***^	1399.06	579	2.416	0.500	0.074	0.878	0.888	0.889	0.666	0.758
Model 4^***^	1560.29	584	2.672	0.053	0.080	0.856	0.867	0.868	0.647	0.745

^***^P < 0.001

## Discussion

The current study aimed to develop a theoretically driven MBBC-6 W and validate its reliability and validity in the Chinese population, which was achieved by following the systematic approach for scale development and comprehensive psychometric validation. In the stage of scale development, the initial 45-item scale was revised and reduced successively to the formal 36-item MBBC-6 W through the presurvey and item analysis. The final scale includes seven dimensions: self-decision behaviour (5 items), self-coping behaviour (5 items), self-control behaviour (5 items), resource coordination behaviour (5 items), resource acquisition behaviour (4 items), breastfeeding operation skills (6 items), and breastfeeding self-perception (6 items). All items of the scale are positive with Likert’s five-point scale, and the scores range from 36 points to 180 points. Meanwhile, the psychometric validation verified that the MBBC-6 W had convincing internal reliability, external reliability, face validity, content validity, structure validity, convergence validity, distinguish validity, and calibration validity, meaning that it is a reliable and valid instrument to assess mothers’ breastfeeding behaviour within 6 weeks postpartum.

The item was an essential part of the scale, and the item analysis was the critical step in scale development. In this study, five methods were used to analyse the capability of items from the perspectives of sensitivity, differentiation, internal consistency, representativeness, importance, and independence. The CV value reflected the sensitivity of items, and the finding represented Item 14: “*For breas*tfeedi*ng, I pay attention to one’s own lifestyle (such as not drinking the strong tea, strong coffee, and alcoholic beverages, not smoking, not taking drugs, etc.)*” had poor sensitivity (CV < 0.15). The possible reason is related to the traditional Chinese puerperium culture, also known as “Zuo Yue Zi”, which deems that lifestyle during puerperium has a long-term impact on maternal health. Thus, most Chinese mothers and their social support system try their best to keep a healthy lifestyle in the puerperium [47]. The correlation coefficient of the scale scores and Item 3 “*I decided to breastfeed is not to meet the expectations of my husband, family or others*” was lower than the standard, meaning Item 3 could not represent the scale. The plausible explanation could be that, with the rise of female consciousness, modern independent women decide to breastfeed their infant because of the benefits for maternal and infant health, rather than to cater to other people [7]. The commonality of Item 27, *“I can recognize the sign of infant hunger accurately and timely*”, was less than the standard, presenting that the importance for scale was poor, which could be caused by maternal different understanding for “infant hunger sign”. Item 28 “*When breastfeeding, I will put the baby’s face close to the breast, and align the tip of baby’s nose at my nipple instead of mouth*” and Item 32 “*For latch well, I support the breast with a C-shape (Place the thumb on top of the breast, and the other four fingers on the chest wall under the breast)*” were deleted due to the lower commonality. The possible reason is the lower completion rate during breastfeeding, which is consistent with the maternal feedback during the daily clinical breastfeeding instruction. Both Item 28 and Item 32 were designed because the abovementioned feeding techniques could help infants latch nipples well. The remaining Item 33, “*During breastfeeding, my infant can always contain the whole nipple, and most of the areola in the mouth*, “ could evaluate the latch results more intuitively. Items 41 “*I think I have sufficient breastmilk to meet infant demand*” and Item 43 “*Breastfeeding makes me feel like a good mother*” were deleted because of the cross-loading, indicating that independence was not recognized. The deletion of Item 43 may be related to it being a comprehensive variable without particularity. The perception of breast milk production is a manifestation of confidence, and the dimension of self-decision behaviour also contains the item evaluating maternal confidence in breastfeeding, which may be the reason why Item 41 belongs to both dimensions [48]. Fortunately, the measuring purpose of Item 40, “*I think breastfeeding made baby gain a healthy weight*”, is similar to Item 41, and it could more objectively evaluate whether breast milk is sufficient.

Reliability, reflecting the internal consistency and stability of the scale, includes internal and external reliability. Cronbach’s α coefficient and the split-half coefficient were used to verify the internal reliability. The results showed that Cronbach’s α coefficient and split-half coefficient of scale were above reference, indicating that the scale has an excellent internal consistency according to the standard classification recommendation [49, 50]. The result showed that the retest coefficient of the scale exceeded the suggested value, meaning that the scale has the capability to obtain a stable result under similar external conditions and has acceptable external reliability [51]. The validity, referring to the ability of the scale to reflect the actual characteristics of the measuring target, consists of face validity, content validity, structure validity, convergence validity, distinguish validity, and calibration validity. In the pilot study, mothers with different education levels, delivery modes, parities, and infant whereabouts were invited to participate in the investigation, ensuring that comprehensive feedback was received from mothers with different characteristics, which provided a good condition to test scale applicability. Among them, most of the mothers (73.3%) had no doubts about items, and eight mothers pointed out some confusion in expression, which was subsequently resolved by the research group referring to their own maternal suggestions; thus, the final revised scale was understandable with good face validity. Seventeen experts reviewed the content validity, and the findings showed that both item-level and scale-level CVI were acceptable, demonstrating that the MBBC-6 W was compatible with the final measuring target. All of the methods often used to extract factors showed that the MBBC-6 W yielded seven common factors, which matched the theoretical model. Moreover, the MBBC-6 W could explain 68.852% of the total variance, confirming that the scale could capture the main characteristics of the mothers’ breastfeeding behaviour within 6 weeks postpartum. On the other hand, the results of CFA showed that, except for TLI being equal to 0.893, all of the remaining fitting indices of the seven-factor model met the statistical requirements. However, the researcher pointed out that the CFI is a valid reference when the TLI is slightly smaller than the standard value [52]. Therefore, although the value of TLI was less than 0.9, the CFI was equal to 0.903, indicating that the seven-factor MBBC-6 W had acceptable structural validity. The factor loadings of items are more significant than the lower limit, indicating that the item-belonging dimension has good convergence validity. Moreover, the value of CR and AVE exceeded the reference, indicating good convergence validity between different dimensions. Distinguishing validity, reflecting the degree of distinction between different dimensions, was analysed by the AVE method and chi-square difference test in this study. Although the distinctive degrees of F1, F2, and F3 were questioned in the results of the AVE method, the chi-square difference test confirmed that the original three-factor model was significantly better than the one-factor model and the two-factor model, indicating that there was a distinction among F1, F2, and F3. The ROC analysis showed that both AUCs were more significant than 0.7 when the scale was used to predict exclusive breastfeeding and any breastfeeding at 42 days, and they were not affected by covariances, suggesting that the scale was a valid tool to predict breastfeeding mode at 42 days. The MBBC-6 W also exhibited specific correlations with the MBFES and BSES-SF, further demonstrating that the MBFES is a valid scale, as breastfeeding behaviour is related to breastfeeding satisfaction and self-efficacy [53].

A new scale named MBBC-6 W was developed and validated in this study, which was designed to measure breastfeeding behaviour among mothers within 6 weeks postpartum. Since there is no specific instrument to evaluate breastfeeding behaviour, the current study has important theoretical and practical implications. In contrast to the existing scales, the scale is conceptually appealing because it directly concentrates on measuring behaviour itself and innovatively focuses on the particular group of mothers within 6 weeks postpartum, which could lay a foundation for evaluation and intervention in future research and clinical practice. However, due to the restrictions of time or conditions, some limitations need to be considered. First, the participants were recruited only in one hospital, and as breastfeeding behaviours are culturally sensitive, the determinants of those behaviours can vary from region to region. Thus, the universality of the scale was limited, and a multicentre study would be built to obtain a scale that would be culturally appropriate and comprehensible for mothers all over the country or even used internationally. Second, the convenience sampling method may affect the sampling representativeness, which could be improved by using random sampling in future research. Third, even though a more extensive sample investigation was implemented, the sample size for EFA and CFA was insufficient because the sample was bisected for the credibility of factor analysis. Subsequent research should continue to expand the sample size to obtain a more stable and reliable model. Fourth, the psychometric verification of MBBC-6 W was based on the classical testing theory (CTT), which has inherent limitations, such as it is challenging to satisfy the assumption of error and accurate score. Further research should use item response theory or multidimensional item response theory to overcome this limitation and provide more information for psychometric testing of the MBBC-6 W. Finally, MBBC-6 W has not yet been applied in clinical practice. The scale should be further used to investigate the current status and potential risk factors to help policy-makers and health workers find problems and formulate corresponding strategies that are conducive to breastfeeding and ultimately achieve the goal of optimal breastfeeding practice.

## Conclusion

Behaviour is the comprehensive manifestation of psychological reaction or movement, and it is necessary to comprehend the particular breastfeeding behaviours that puerperium mothers gain due to the particularity and importance of the puerperium. The newly developed MBBC-6 W is a reliable and valid instrument for assessing the breastfeeding behaviour of Chinese mothers within 6 weeks postpartum, making it possible for us to identify the current state of maternal breastfeeding behaviour and the details that should be improved. Further research is needed to explore the specific strategies that can promote best practices in breastfeeding.

## Data Availability

All data generated or analysed during this study are included in this published article.
